# Dysregulated Methylation Patterns in Exon IV of the Brain-Derived Neurotrophic Factor (BDNF) Gene in Nicotine Dependence and Changes in BDNF Plasma Levels During Smoking Cessation

**DOI:** 10.3389/fpsyt.2022.897801

**Published:** 2022-06-28

**Authors:** Kerim Abdelkhalek, Mathias Rhein, Maximilian Deest, Vanessa Buchholz, Stefan Bleich, Ralf Lichtinghagen, Benjamin Vyssoki, Helge Frieling, Marc Muschler, Phileas Johannes Proskynitopoulos, Alexander Glahn

**Affiliations:** ^1^Department of Psychiatry, Social Psychiatry and Psychotherapy, Hannover Medical School, Hannover, Germany; ^2^Department of Clinical Chemistry, Hannover Medical School, Hannover, Germany; ^3^Board of Trustees for Psychosocial Services in Vienna, Vienna, Austria

**Keywords:** tobacco dependence, addiction - smoking, addiction, epigenetic, addict behavior

## Abstract

**Introduction:**

Several studies reported dysregulated protein levels of brain-derived neurotrophic factor (BDNF) in smokers and during cessation. However, the epigenetic regulation of the BDNF gene has not yet been investigated. We measured the plasma levels of BDNF and the epigenetic regulation of exon IV of the BDNF gene in smokers compared to healthy controls over a cessation period of 14 days.

**Method:**

We measured BDNF plasma levels and BDNF promoter methylation in 49 smokers and 51 non-smokers at baseline, day 7, and day 14 of smoking cessation. Mean methylation levels of 11 Cytosine Guanosine dinucleotides of exon IV of the BDNF gene were determined *via* bisulfite sequencing.

**Results:**

BDNF plasma and methylation levels were significantly lower in healthy controls when compared with smokers across all time points. BDNF levels for smokers decreased significantly during the cessation period. Comparing the sexes, female smokers showed significantly lower plasma BDNF levels than healthy controls at baseline and over 14 days of cessation. Male and female smokers showed significantly higher mean methylation rates than non-smokers at baseline. In male smokers, mean methylation levels decreased significantly during the cessation period.

**Conclusion:**

Our findings replicate the findings of previous studies that BDNF plasma levels are altered in smokers. Furthermore, BDNF expression and gene methylation are altered during the first 14 days of cessation. Our novel findings of dysregulated methylation patterns in exon IV of the BDNF gene further support the thesis that BDNF plays a role in nicotine dependence.

## Introduction

Cigarette smoking is one of the leading preventable causes of related chronic diseases and deaths worldwide (1). Nicotine is the main psychoactive component of tobacco that affects many neurotransmitter systems and other factors such as brain-derived neurotrophic factor (BDNF) (2). BDNF, a member of the neurotrophin family (3), is abundantly expressed in the central and peripheral nervous systems (4, 5) as well as peripheral tissue such as platelets (6). It is involved in many critical neuronal processes like developing and regulating neuro (7)-, glio (8)- and synaptogenesis (9). As a promoter of neurite growth, it fosters physiological neuronal system development (7).

Brain-derived neurotrophic factor also modulates diverse neurotransmitter systems like glutamatergic, dopaminergic, and serotonergic systems (10). Peripheral BDNF can be analyzed in plasma and serum and some studies reported a positive correlation with brain BDNF (11, 12). Thus, depending on the context, changes in peripheral BDNF can, to a limited extent, be used as surrogates of brain changes. Earlier studies supported evidence that BDNF plays a crucial role in several substance addictions, such as alcohol, cocaine, and methamphetamine addiction (13). In the context of nicotine dependence, several animal studies suggest that BDNF is functionally involved (2). Previous studies in humans showed that peripheral BDNF levels are altered in smokers compared to non-smokers. While the first two studies showed a decrease in BDNF in smokers (14, 15), all subsequent studies observed an increase in BDNF protein levels in smokers (16–18).

Furthermore, studies have also investigated methylation of BDNF promoter I in major depressive disorder, showing an association between neurocognitive performance and two BDNF SNPs, while methylation levels mediated this effect at specific sites of promoter I (19). In another study, higher BDNF methylation levels at exon I and exon IV were associated with major depression (20). According to Ikegame et al., patients suffering from mental disorders generally show decreased neural BDNF levels, which are often – but not always – associated with DNA methylation at specific BDNF promoter regions (21). Hence, we assumed that changes in plasma BDNF levels would be related to changes in the methylation status of the BDNF promoters. However, to the best of our knowledge, no studies have investigated the epigenetic regulation of the BDNF gene in the context of nicotine dependence and smoking cessation. We hypothesized that plasma BDNF levels would be associated with methylation levels at exon IV promoter of BDNF and that changes in protein levels would be associated with changes in methylation levels over the cessation period. This study investigates plasma BDNF levels and methylation rates of exon IV of the BDNF gene in smokers compared to healthy non-smokers at baseline and over a cessation period of 14 days.

## Materials and Methods

This study adhered to the Declaration of Helsinki and was approved by the local Ethics Committee of Hannover Medical School (approval number: 6695). We included 49 smokers with nicotine dependence as defined by the International Classification of Diseases and Diagnostics (ICD-10) and Statistical Manual of Mental Disorders (DSM IV) (Table 1). As controls, 51 healthy non-smokers were recruited. All participants in this study gave written informed consent. Exclusion criteria were concomitant psychiatric illness, other substance or alcohol abuse or dependence, cerebral ischemia, cerebral hemorrhage, epilepsy, cardiovascular and renal diseases, age under 18 years, pregnancy, and nicotine replacement therapy. Inclusion criteria were age 18–65 years and current smoker (minimum seven cigarettes per week or one cigarette a day). The severity of nicotine addiction was measured using the Fagerström-Test, while craving was assessed using the Questionnaire of Smoking Urges (QSU).

**Table 1:** Demographics.

		**Age**			**BMI**			**Cigarettes/Day**			**QSU score**			**Fagerström**		
	**Count (*N*)**	**Mean**	**SD**	***N***	**Mean**	**SD**	***N***	**Mean**	**SD**	***N***	**Mean**	**SD**	***N***	**Mean**	**SD**	***N***
**Controls**
Male	25	25.17	7.54	24	24.09	2.70	25									
Female	26	27.42	7.12	26	22.17	3.20	26									
**Smokers**
Male	29	29.56	10.08	25	26.61	4.12	25	11.40	7.26	25	70	28	29	2	2	29
Female	20	33.44	9.56	18	23.53	3.69	20	12.72	7.89	18	64	17	20	3	2	20

*Some smokers and controls did not report age and BMI. For our Analysis of promoter methylation and BDNF plasma levels we included all samples with valid values (49 smokers vs. 51 controls)*.

*BMI, body mass index; N, number; QSU, questionnaire of smoking urges; SD, standard deviation*.

All smokers underwent a detailed physical examination, routine laboratory testing, and urine drug screening. Fasting blood samples and cotinine to check for relapse were drawn from nicotine-dependent smokers and the controls on days 1, 7, and 14 (t0, t7, t14) of abstinence between 8:00 and 10:00 a.m. We choose a period of 2 weeks since withdrawal symptoms tend to peak during the first week and can last up to four more weeks (22). In a different study, the authors have observed changes in mean methylation of the BDNF promoter over 14 days in smokers during alcohol withdrawal therapy (23). All blood samples were anti-coagulated with sodium EDTA. Plasma was separated in a centrifuge at 4,000 g, and the aliquots were stored at −80°C. BDNF plasma levels were measured using the Quantikine Total BDNF ELISA (Cat# DBNT00, R&D Systems, Minneapolis, USA). As the sample count exceeded assay size, we decided to measure samples from equal time points on one plate, applying the same standard to every measurement. DNA for methylation analysis was extracted from blood using the NucleoMag 200 kit (Macherey&Nagel, Düren, Germany). Bisulfite conversion and purification were performed using the EpiTect®96 Bisulfite Kit 142 (Qiagen, Hilden, Germany) following the manufacturer's recommendations. A detailed protocol of bisulfite sequencing and determination of methylation rates is provided in Supplemental File 1.

### BDNF Exon IV

The BDNF gene has 11 exons and nine functional promoters (24). We investigated BDNF exon IV since it has been extensively studied in psychiatric research, mainly in the context of depression (25).

### Statistical Analysis

All statistical analyses were performed using the Statistical Package for Social Sciences 26 (SPSS, IBM, Armonk, NY, USA). GraphPad Prism 9 (San Diego, California, USA) was used for data illustration. As normality was not present in both methylation data and ELISA measurements, we used the Kruskal–Wallis Test to test for differences across controls and smokers. For pairwise comparisons, we performed Dunn's *post hoc* test with Bonferroni correction for multiple comparisons in independent samples to compare healthy controls with smokers. To measure changes across time points in the smokers' groups we used Friedman's Test for dependent samples, followed by Dunn's Test for pairwise comparison while controlling for multiple testing using Bonferroni correction. For bivariate correlations, we applied the Spearman method as a non-parametric option, accordingly.

## Results

### BMI

BMI was significantly lower in controls compared with smokers across all time points (*H* (3) = 13.45, *P* = 0.004). Furthermore, BMI was significantly lower in controls when compared with t0, t7, t14 (*H* (1) = −29.76, *P* = 0.04; *H* (1) = −33.05, *P* = 0.015; *H* (1) = −32.24, *P* = 0.014). In males, BMI was significantly lower in controls when compared to smokers across time points (*H* (3) = 10.55, *P* = 0.014), while there was no difference for females. Furthermore, BMI was significantly lower in male controls when compared with t0, and t14 (*H* (1) = −21.87, *P* = 0.046; *H* (1) = −21.92, *P* = 0.042).

### Peripheral BDNF Levels

BDNF plasma levels were significantly lower in controls when compared to smokers across timepoints (*H* (3) = 16.56, *P* = 0.001). Furthermore, BDNF levels were significantly lower in controls when compared with t0, t7, t14 (*z* = −42.64, *P* = 0.001; *z* = −35.50, *P* = 0.011; *z* = −31.30, *P* = 0.035). In smokers, no significant change was observed during withdrawal.

**Figure 1:**
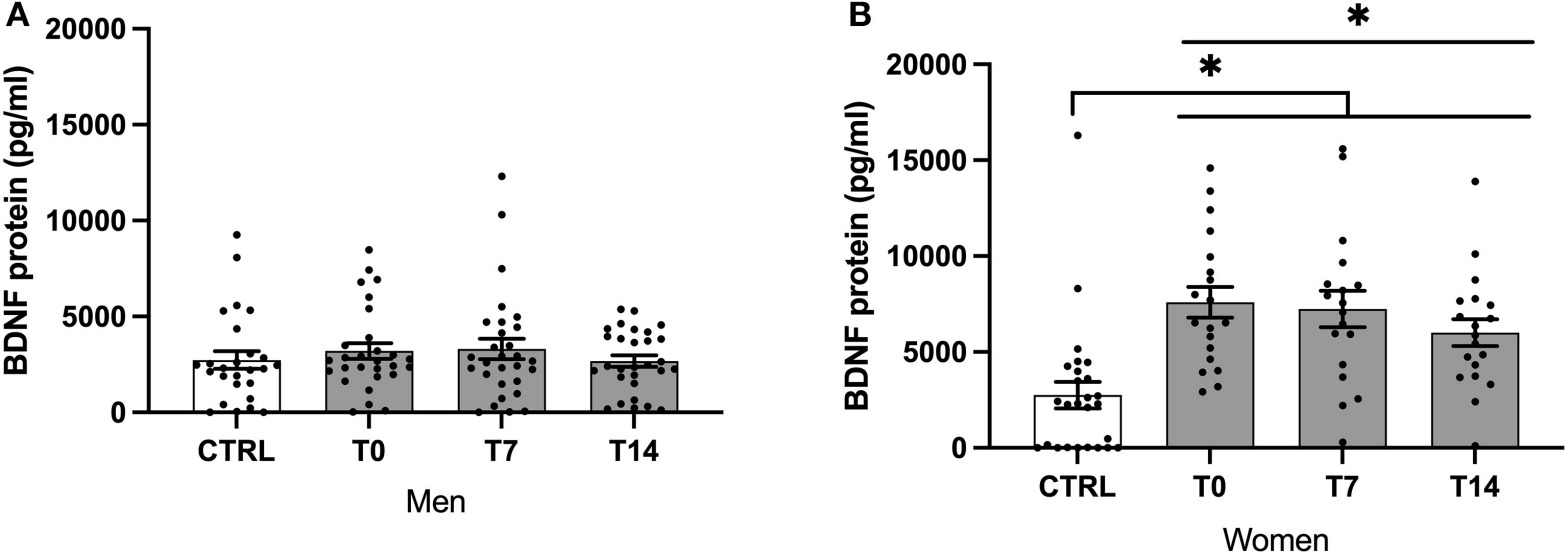
BDNF protein levels of male **(A)** and female **(B)** smokers at baseline, day 7 and day 14 of cessation vs. healthy controls. BDNF, brain-derived neurotrophic factor; CTRL, healthy controls; T0, first day of cessation; T7, day 7 of cessation; T14, day 14 of cessation. Significant differences are indicated by asterisks (*P* ≤ 0.05*).

In females, BDNF plasma levels were significantly lower in controls when compared to smokers across timepoints (*H* (3) = 26.79, *P* < 0.001). While there was no difference for males, BDNF levels were significantly lower in female controls when compared with female smokers at t0, t7, t14 (*z* = −33.09, *P* < 0.001; *z* = −29.17, *P* = 0.001; *z* = −26.82, *P* = 0.002; see Figure 1).

BDNF levels decreased significantly during the cessation period (χ^2^ (2) = 7.46, *P* = 0.024). Using pairwise comparison, BDNF levels were significantly lower at T14 compared with T0 (*P* = 0.04; see Figure 1).

In females, BDNF levels decreased significantly during the cessation period (χ^2^ (2) = 6.10, *P* = 0.047). There was no significant difference when comparing different timepoints after the Bonferroni correction (see Figure 1).

### Methylation Analysis

As a first step, we investigated differences in methylation levels at specific CpG islands of the promoter region of exon IV. Comparison of specific CpGs did reveal no significant differences between healthy controls and smokers (see Supplementary Figure S1). We further compared mean methylation of exon IV promoter for the two groups and genders. Mean methylation levels were significantly lower in controls when compared to smokers across time points (*H* (3) = 21.07, *P* < 0.001). Furthermore, mean methylation significantly lower in controls when compared with t0, t7, t14 (*z* = −49.56, *P* < 0.0001; *z* = −30.55, *P* = 0.044; *z* = −38.59, *P* = 0.004). In smokers, no significant change was observed during withdrawal.

**Figure 2:**
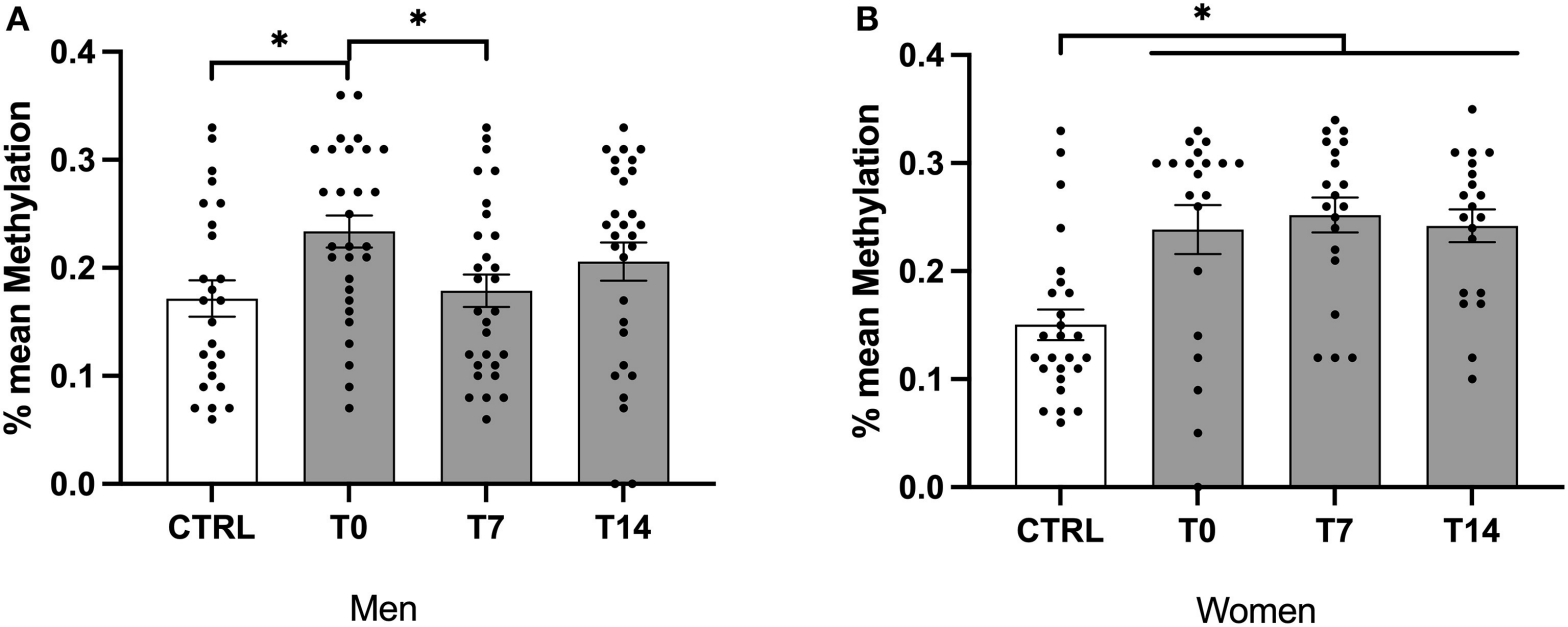
Mean methylation levels of exon IV in BDNF gene of male and female smokers at baseline, day 7 and day 14 of cessation vs. healthy controls. BDNF, brain-derived neurotrophic factor; CTRL, healthy controls; T0, first day of cessation; T7, day 7 of cessation; T14, day 14 of cessation. Significant differences are indicated by asterisks (*P* ≤ 0.05*). BDNF protein levels of male **(A)** and female **(B)** smokers at baseline.

In males and females, mean methylation levels were significantly lower in controls when compared to smokers across time points (*H* (3) = 9.65, *P* = 0.022; *H* (3) = 18.63, *P* < 0.001, respectively). Furthermore, mean methylation levels were significantly lower in female controls when compared with female smokers at t0, t7, t14 (*z* = −24.09, *P* = 0.006; *z* = −27.69, *P* = 0.001; *z* = −22.76, *P* = 0.012) and in male controls when compared with male smokers at t0 (*H* (1) = −23.690, *P* < 0.05; Figure 2).

Across smokers, there was no significant change in mean methylation levels during the cessation period. In male smokers, mean methylation levels decreased significantly during the cessation period (χ^2^ (2) = 6.07, *P* = 0.048). Furthermore, using the non-parametric 'Dunn's test, mean methylation was significantly lower at T7 when compared with T0 (*P* = 0.045). Of note, there was no difference between T0 and T14 as well as between T7 and T14 (Figure 2).

### Association Between Craving and Mean Methylation

We used Spearman's correlation analysis to analyze a relationship between mean methylation, BDNF plasma levels, and questionnaire of smoking urges (QSU) scores at all three time points. Here, we found a positive association between the QSU (subtest 2) and the total QSU score with mean methylation (*r*(145) = 0.228, *P* = 0.06; *r*(145) = 0.191, *P* = 0.02, respectively).

### Association Between Addiction Severity and Methylation

We used the Fagerström test for nicotine dependence (FTND) to assess how BDNF promoter methylation and protein levels related to addiction severity (26). Nonparametric correlation analysis revealed a significant correlation of both methylation (*r* (47) = 0.322, *P* = 0.024) and protein levels (*r* (47) = 0.362, *P* = 0.011) at time point t14. Plotting these correlations, however, did not reveal a significant association between addiction severity and methylation (*r* > 0.098; Supplementary Figure S2). Also, using the classification from the previous study [1 (FTND < 3), 2 (FTND = 3–4), 3 (FTND > 4)], groups did not differ significantly when put in relation to either protein levels or mean promoter fragment methylation (data not shown).

### Correlation Between BMI, Mean Methylation and BDNF Levels

Using Spearman's Correlation Coefficient, BMI showed no association with BDNF plasma levels and mean methylation across the whole sample (controls, smokers at t0, t7, and t14).

## Discussion

As we expected BDNF levels to be altered by addiction and within the cessation period of 14 days, we investigated the effect of smoking cessation on plasma BDNF levels and methylation of exon IV of the BDNF gene. BDNF plasma and methylation levels were significantly higher in smokers when compared with controls across all time points. Mean methylation was significantly higher in smokers when compared with healthy controls across all time points. Also, female smokers showed significantly lower plasma BDNF levels than healthy controls at baseline and over 14 days of cessation.

### Dysregulation of BDNF in Nicotine Dependence

Our findings indicate that BDNF could be dysregulated in smokers, while there was no significant change in methylation rates during cessation in both sexes across all time points. We found significant differences in males when comparing day 1 and 7 methylation percentages. Meanwhile, we observed significantly decreasing BDNF protein levels, even though we found no corresponding change in methylation, indicating a complex regulation of which methylation is only one contributing influence. In male smokers, mean methylation levels decreased significantly during the early cessation period (day 1 and 7) and then regressed to the first value. In female smokers, BDNF levels decreased significantly over the whole cessation period. Taken together, our results suggest that BDNF plasma expression and mean methylation are influenced by smoking as well as smoking cessation.

Interestingly, we found a positive association between mean methylation levels and craving across all time points. Even though this finding points to a possible influence of methylation levels on craving, the effect sizes are only small. We would therefore interpret this very interesting result as a trend that needs future investigations.

Furthermore, BDNF could be involved in the steps of a cascade of several dysregulated pathways involved in nicotine dependence. Several studies investigated the effect of smoking on peripheral BDNF levels (14–18), while most studies showed an increase of peripheral BDNF in smokers compared to non-smokers, which is in line with our findings of elevated BDNF levels in smokers. Further investigation is needed to validate methylation and plasma expression levels of BDNF over a longer period than 14 days, especially to investigate whether BDNF levels approach levels of non-smokers.

### Influence of BMI on BDNF

There is evidence that plasma BDNF levels vary in relation to body weight in females (27). In the present study, BMI levels were not associated with plasma BDNF levels and significantly lower in controls compared with smokers. Since our assumptions for performing parametric tests were not met, we could not perform an analysis of covariance to identify a possible influence of BMI on BDNF levels. In line with previous findings, one possible explanation is that BMI differences could explain our varying results. However, since several factors influence BMI while BDNF seems to be associated with addictive behavior predominantly, it is possible that the changes could be due to cessation. This is also highlighted by the fact that BDNF levels decreased significantly during the observation period.

### Factors Influencing BDNF Expression

Increasing evidence has shown that sociodemographic variables and lifestyle factors such as food and alcohol intake influence peripheral BDNF levels (28). In one study on BDNF, the authors concluded that future studies should consider correcting for the time of blood withdrawal, storage, urbanicity, age, sex, smoking status and food and alcohol intake (28). In the present study, we accounted for some of the mentioned variables by performing blood withdrawal and immediately (>2 h) storing all samples in a specific manner (s. methods). As we could not include the potential influence of age and sex in the main analysis, we validated the role of these variables for methylation by correlation analysis and could not see an influence (data not shown). Furthermore, we did not specifically assess urbanicity, food, or alcohol intake. Regarding sex, one study on the association between BDNF levels and major depressive disorder reported that, in females, BDNF levels decline with age while remaining stable in males. Furthermore, after controlling for gender and age, the assays showed lower serum BDNF levels being associated with higher depression scores. Interestingly, in this study the effects remained significant after correction for withdrawal time and smoking (29). Here we found peripheral BDNF levels to decrease during the cessation in females but not in males. In conjunction with the discussed evidence, our results suggest possible gender-specific differences. Due to the non-parametric nature of our data, we did not conduct further analysis to identify a possible influence of age and gender on protein or methylation levels. Respectively, one study has argued that BDNF levels are generally not normally distributed (30), and thus Gass and Hellweg (31) conclude that small studies using parametric tests could therefore be misleading.

### BDNF Methylation or Expression and Addiction Severity

Both QSU and FTND show slight aspects of association upon initial correlation but fail to reveal substantial predictive value for both addiction severity and craving. This is in part due to the small cohort, where stratification is limited. With BDNF changes being at the periphery of the regulatory processes that are involved in reward circuitry regulation, variance is likely to be increased and therefore requiring bigger cohorts to justify reliable interpretation.

### The Role of Peripheral BDNF Levels for Regulation in the Brain

From studies in rodents, peripheral and brain BDNF protein levels appear to correlate (11, 12). For the human brain, levels are different in distinct brain areas (32) and research has shown both evidence supporting and contradicting a correlation between central and peripheral BDNF-levels (33). We, therefore, do not associate methylation and peripheral expression levels with the situation in the addicted brain. Of note, circulating BDNF levels have been suggested to be associated with cognitive function, with lower levels being found in patients with amnestic mild cognitive impairment (34). Thus, differences in peripheral levels could be partly explained by molecular differences leading to cognitive function. This is important since a prospective study by Vermeulen et al. (35) on the association between smoking behavior and cognitive function in patients with psychosis, their siblings, and healthy controls has shown that smoking is associated with poorer cognitive function in each group compared with nonsmoking. The mean age in our study was similar to that in this study by Vermeulen et al. (35), highlighting this critical factor influencing BDNF levels. In contrast, a recent review concluded that BDNF is dysregulated in many pathological conditions and cannot be regarded as a valid biomarker but a marker related to mnemonic symptoms' occurrence or progression (32).

Concerning peripheral BDNF levels in major depression, one review has highlighted platelet function as a possible confounding factor influencing BDNF measurement, with platelets being the major source of peripheral BDNF (33). Since cigarette smoke is well known to affect platelet function (36–38), a recent study has shown that platelet-derived BDNF regulates tissue factor expression and that cigarette smoke stimulates pro-atherothrombotic states (39). Also, human platelets treated with an aqueous extract of cigarette smoke released BDNF in a dose-dependent manner (39). Therefore, smoking cessation might influence platelet function and, in consequence, BDNF-level expression, while the kinetics of these effects remain unclear. The significantly higher BDNF levels we found in smokers could be caused by the effect of smoking on platelet function. Furthermore, it seems possible that the normalization of platelet function could partly explain the significant change in BDNF levels during cessation groups.

### Peripheral BDNF as a Potential Biomarker

As reduced BDNF is associated with several mental disorders, its role as a possible biomarker has been studied extensively (31). Several studies have shown that BDNF levels are decreased in mental disorders such as depression (40), schizophrenia (41, 42), anxiety disorders (43), and cognitive impairment (44), to name a few. According to Gass and Hellweg (31), one explanation could be that affective disorders share the common contributing and sustaining factor stress which is well known to influence BDNF levels, regulation, and signaling (45–48). One recent and rigorous review has proposed a BDNF stress-sensitivity hypothesis. The authors argue that disruption of endogenous BDNF activity by factors potentiates sensitivity to stress and vulnerability to stress-inducible illnesses and propose mechanisms by which BDNF may induce plasticity to promote fear and trauma while enabling adaptive plasticity during extinction learning (49). It is fair to conclude that alterations in BDNF levels are neither disease nor treatment specific since stress is a major factor in mental disorders. However, the differences observed in our study could partly be explained by smoking cessation being the altered factor in smokers, even though we were not able to control for all relevant confounding variables.

### Limitations

The present study has several limitations. The reported BDNF levels could partly be explained by differences in BMI, since we could not perform an analysis of covariance. Nonetheless and of note, BMI did not correlate with BDNF plasma expression, suggesting that the differences found could be explained by smoking cessation. One explanation could be the relatively small sample size and the fact that we used non-parametric tests with lower power than parametric tests. Since we did not assess platelet count, we cannot perform further analysis to identify a possible influence of platelet count on BDNF expression levels and promoter methylation. In addition, we were only able to control for some of the previously mentioned confounding variables (Section Factors Influencing BDNF Expression).

Furthermore, we only investigated exon IV as being the most prevalent target in psychiatric research. While it is also worth noting that two studies reported opposing results, namely decreased BDNF levels in smokers, those studies also showed an increase of BDNF levels over 2–3 months (15). In contrast to these findings, we did not observe any convergence of BDNF levels with controls, supposedly due to the short observation period of 14 days of cessation. Since physical nicotine detoxification can last around 4 weeks and be associated with psychiatric side effects, it is essential to investigate BDNF levels and promoter methylation during a more extended follow-up period while correlating changes in methylation and plasma levels with psychometric parameters. In our study we put emphasis on those smokers that remained abstinent over the period of 14 days. Thus, we were not able to differentiate between abstainers and relapsers to analyze a possible role of BDNF as relapse marker, which should be done in future studies.

## Conclusion

Our findings replicate those of previous studies that peripheral BDNF is elevated in smokers. Also, BDNF levels decreased during the short cessation period. Our novel findings of dysregulated methylation patterns in exon IV of the BDNF gene further support the hypothesis that epigenetic regulation of BDNF plays a role in nicotine dependence in a gender-dependent manner. Should further studies confirm these results, measuring BDNF promoter IV methylation to determine addiction severity and relapse probability could be a sensitive readout for the clinical application that could enhance therapy and indicate the efficacy of relapse prevention.

## Data Availability Statement

The original contributions presented in the study are included in the article/Supplementary Material, further inquiries can be directed to the corresponding author.

## Ethics Statement

The studies involving human participants were reviewed and approved by Ethics Committee, Medical School Hannover, 30625 Hannover. The patients/participants provided their written informed consent to participate in this study.

## Author Contributions

AG and MM planned and carried out the study. RL analyzed the BDNF ELISA measurements. VB and MR generated the methylation data. KA, PP, MD, and MR analyzed the data and wrote the manuscript. SB, BV, AG, MR, HF, KA, MM, PP, and MD critically revised and contributed to the manuscript. All authors contributed to the article and approved the submitted version.

## Funding

MD is supported by PRACTIS – Clinician Scientist Program of Hannover Medical School, funded by the German Research Foundation (DFG, ME 3696/3-1). AG was supported by HiLF (Hochschulinterne Leistungsförderung).

## Conflict of Interest

The authors declare that the research was conducted in the absence of any commercial or financial relationships that could be construed as a potential conflict of interest.

## Publisher's Note

All claims expressed in this article are solely those of the authors and do not necessarily represent those of their affiliated organizations, or those of the publisher, the editors and the reviewers. Any product that may be evaluated in this article, or claim that may be made by its manufacturer, is not guaranteed or endorsed by the publisher.

## Supplementary Material

Supplementary Figure S1 Detailed CpG methylation for the analyzed promoter fragment of the BDNFIV exon. **(A)** Overview of mean methylation (*y*-axis) per CpG (*x*-axis) for Controls (blue) and patient timepoints (T0-green, T7-dark red, T14-bright red). **(B,C)** Provide the gender-specific display of all CpGs.As the general trend of the majority of CpGs is represented in the mean values for the whole fragment, we refrained from looking at a detailed comparison of certain positions for analysis. Error bars are ±1 SEM.

Supplementary Figure S2 Comparison of methylation and protein levels with the Fagerström questionnaire results (FTND). **(A)** Nonparametric Spearman Correlation of Protein levels (BDNF_E), mean methylation (mean_meth), FTND and FTND binning for groups defined by Lesch et al. (>3 = 1, 3–4 = 2, >4 = 3, FTND Lesch). **(B)** Display of mean methylation values for the three Lesch FTND groups divided by timepoint (T0-green, T7-dark red, T14-bright red). **(C–H)** Plot of mean methylation **(C–E)** and protein levels **(F–H)** against the FTND questionnaire results. Regression means with R square values for total trend and according to sex are given for each timepoint.
