# Supplementary figures and images for: Dysregulated Methylation Patterns in Exon IV of the Brain-Derived Neurotrophic Factor (BDNF) Gene in Nicotine Dependence and Changes in BDNF Plasma Levels During Smoking Cessation
Source: Front Psychiatry. 2022 Jun 28;13:897801. doi: 10.3389/fpsyt.2022.897801 (PMC9273814; doi:10.3389/fpsyt.2022.897801)

Figure S1

A

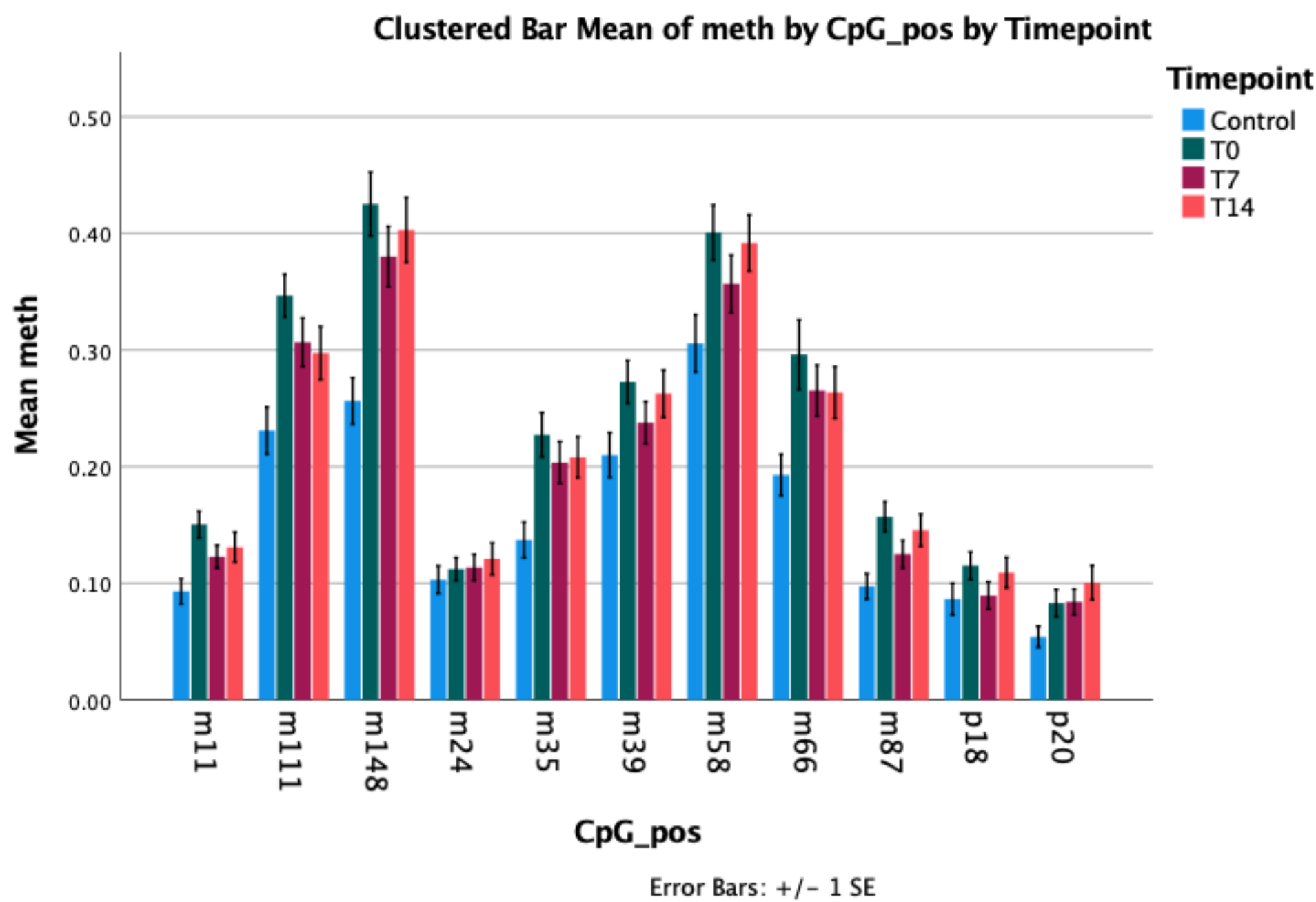

B

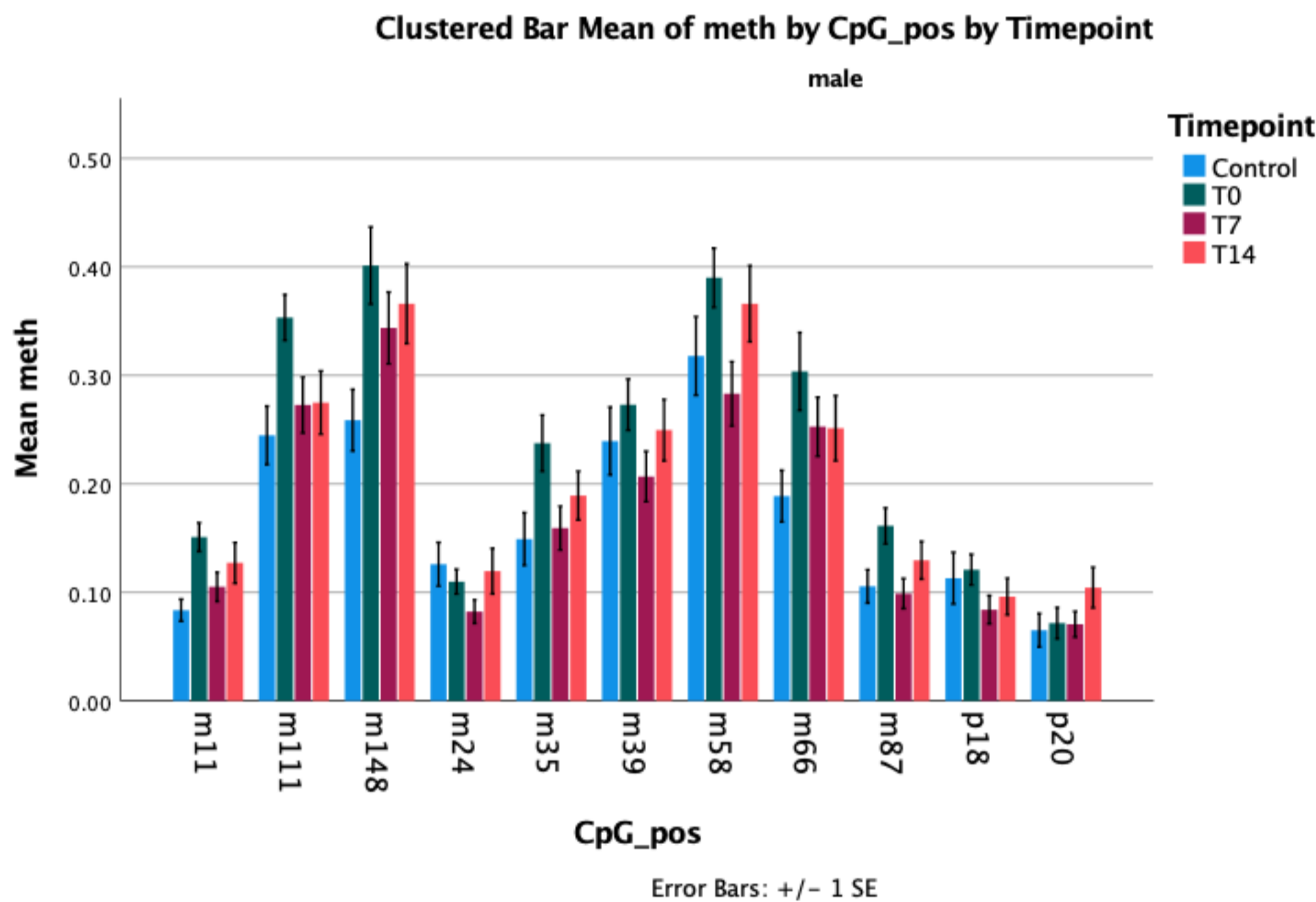

C

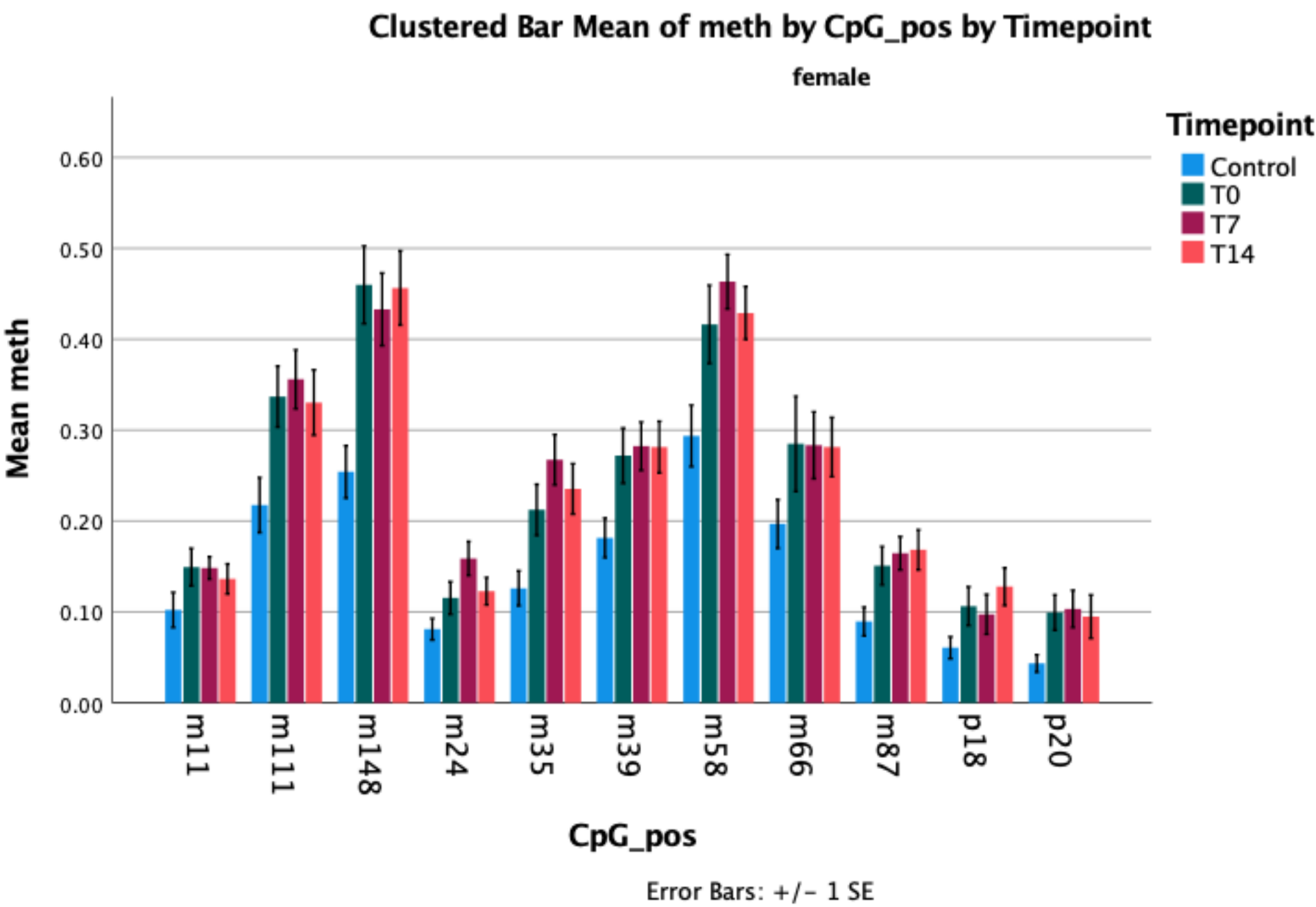

Supplement: Supplementary Figure S1 — Detailed CpG methylation for the analyzed promoter fragment of the BDNFIV exon. (A) Overview of mean methylation (y-axis) per CpG (x-axis) for Controls (blue) and patient timepoints (T0-green, T7-dark red, T14-bright red). (B,C) Provide the gender-specific display of all CpGs.As the general trend of the majority of CpGs is represented in the mean values for the whole fragment, we refrained from looking at a detailed comparison of certain positions for analysis. Error bars are ±1 SEM. [file Data_Sheet_1.PDF]
