# Supplementary material for: Dysregulated Methylation Patterns in Exon IV of the Brain-Derived Neurotrophic Factor (BDNF) Gene in Nicotine Dependence and Changes in BDNF Plasma Levels During Smoking Cessation
Source: Front Psychiatry. 2022 Jun 28;13:897801. doi: 10.3389/fpsyt.2022.897801 (PMC9273814; doi:10.3389/fpsyt.2022.897801)

Figure S2

A

Correlations

Timepoint				FTND	FTND Lesch	BDNF_E	mean_meth
1 T0	Spearman's rho	FTND	Correlation Coefficient	1.000	.928**	.181	.096
			Sig. (2-tailed)	.	.000	.214	.510
			N	49	49	49	49
		FTND Lesch	Correlation Coefficient	.928**	1.000	.148	.034
			Sig. (2-tailed)	.000	.	.311	.817
			N	49	49	49	49
		BDNF_E	Correlation Coefficient	.181	.148	1.000	-.115
			Sig. (2-tailed)	.214	.311	.	.431
			N	49	49	49	49
		mean_meth	Correlation Coefficient	.096	.034	-.115	1.000
			Sig. (2-tailed)	.510	.817	.431	.
			N	49	49	49	49
2 T7	Spearman's rho	FTND	Correlation Coefficient	1.000	.928**	.269	.201
			Sig. (2-tailed)	.	.000	.061	.167
			N	49	49	49	49
		FTND Lesch	Correlation Coefficient	.928**	1.000	.242	.177
			Sig. (2-tailed)	.000	.	.094	.225
			N	49	49	49	49
		BDNF_E	Correlation Coefficient	.269	.242	1.000	.303*
			Sig. (2-tailed)	.061	.094	.	.035
			N	49	49	49	49
		mean_meth	Correlation Coefficient	.201	.177	.303*	1.000
			Sig. (2-tailed)	.167	.225	.035	.
			N	49	49	49	49
3 T14	Spearman's rho	FTND	Correlation Coefficient	1.000	.928**	.362*	.322*
			Sig. (2-tailed)	.	.000	.011	.024
			N	49	49	49	49
		FTND Lesch	Correlation Coefficient	.928**	1.000	.338*	.244
			Sig. (2-tailed)	.000	.	.018	.091
			N	49	49	49	49
		BDNF_E	Correlation Coefficient	.362*	.338*	1.000	.162
			Sig. (2-tailed)	.011	.018	.	.266
			N	49	49	49	49
		mean_meth	Correlation Coefficient	.322*	.244	.162	1.000
			Sig. (2-tailed)	.024	.091	.266	.
			N	49	49	49	49

**. Correlation is significant at the 0.01 level (2-tailed).

*. Correlation is significant at the 0.05 level (2-tailed).

B

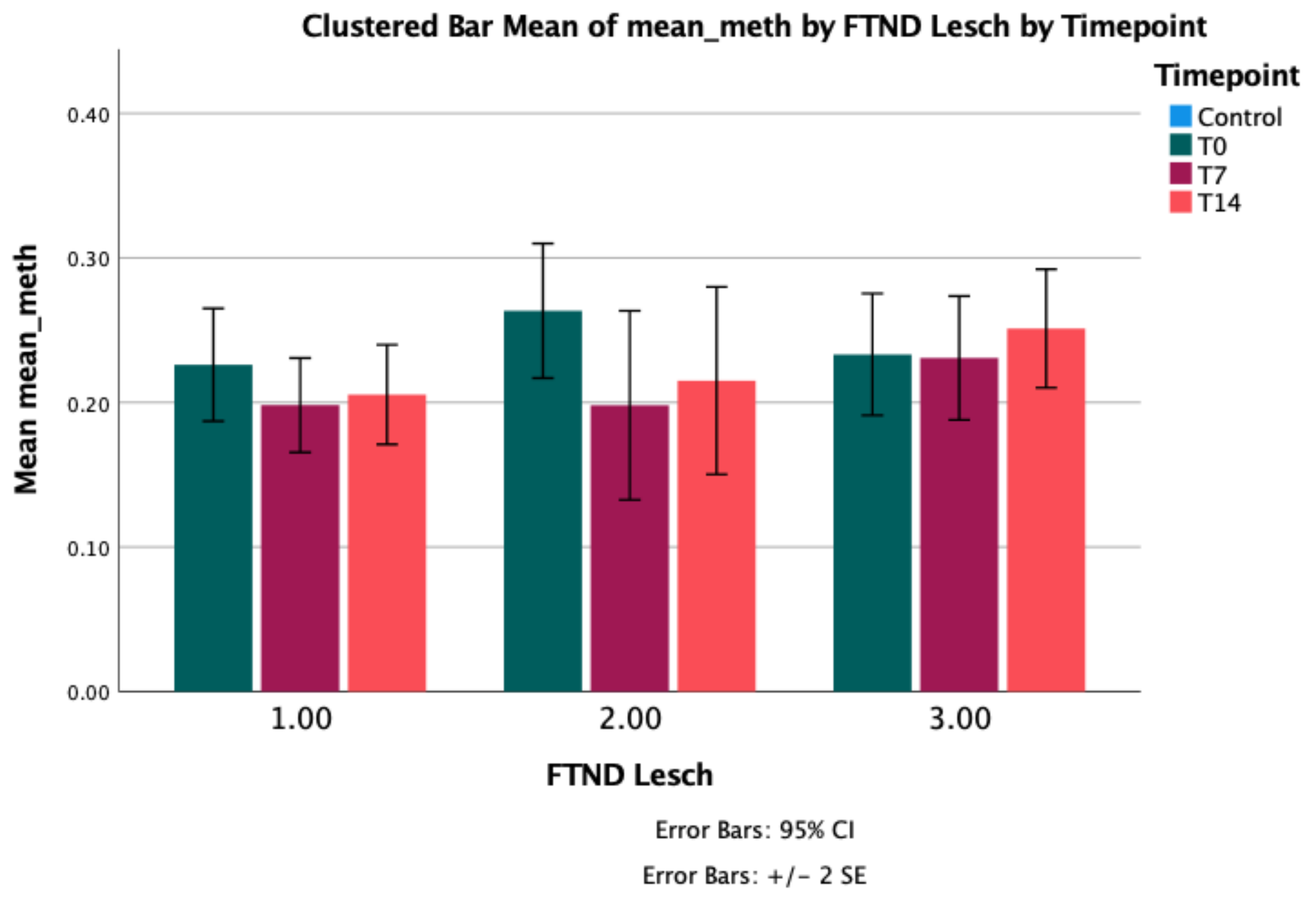

C

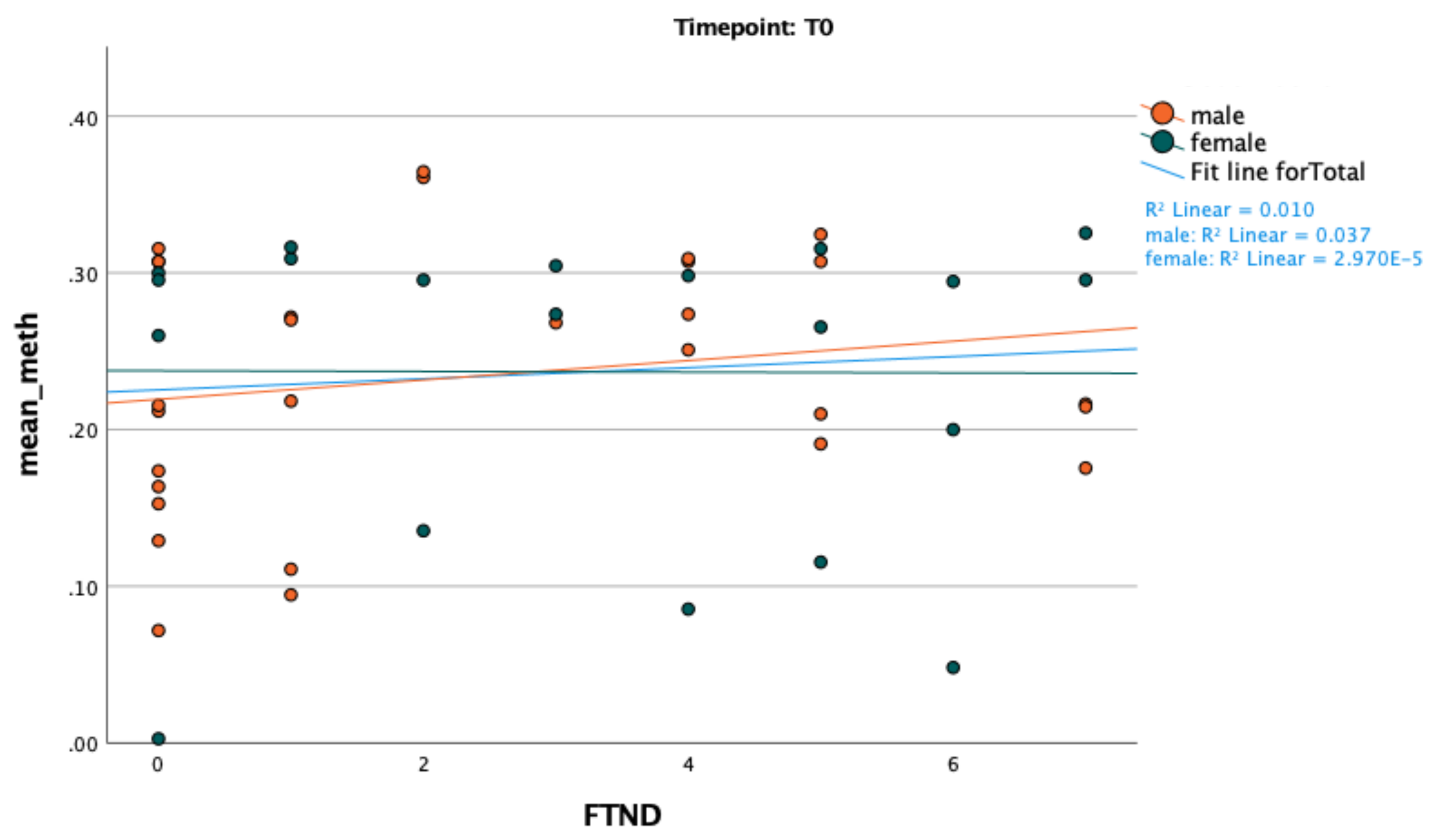

D

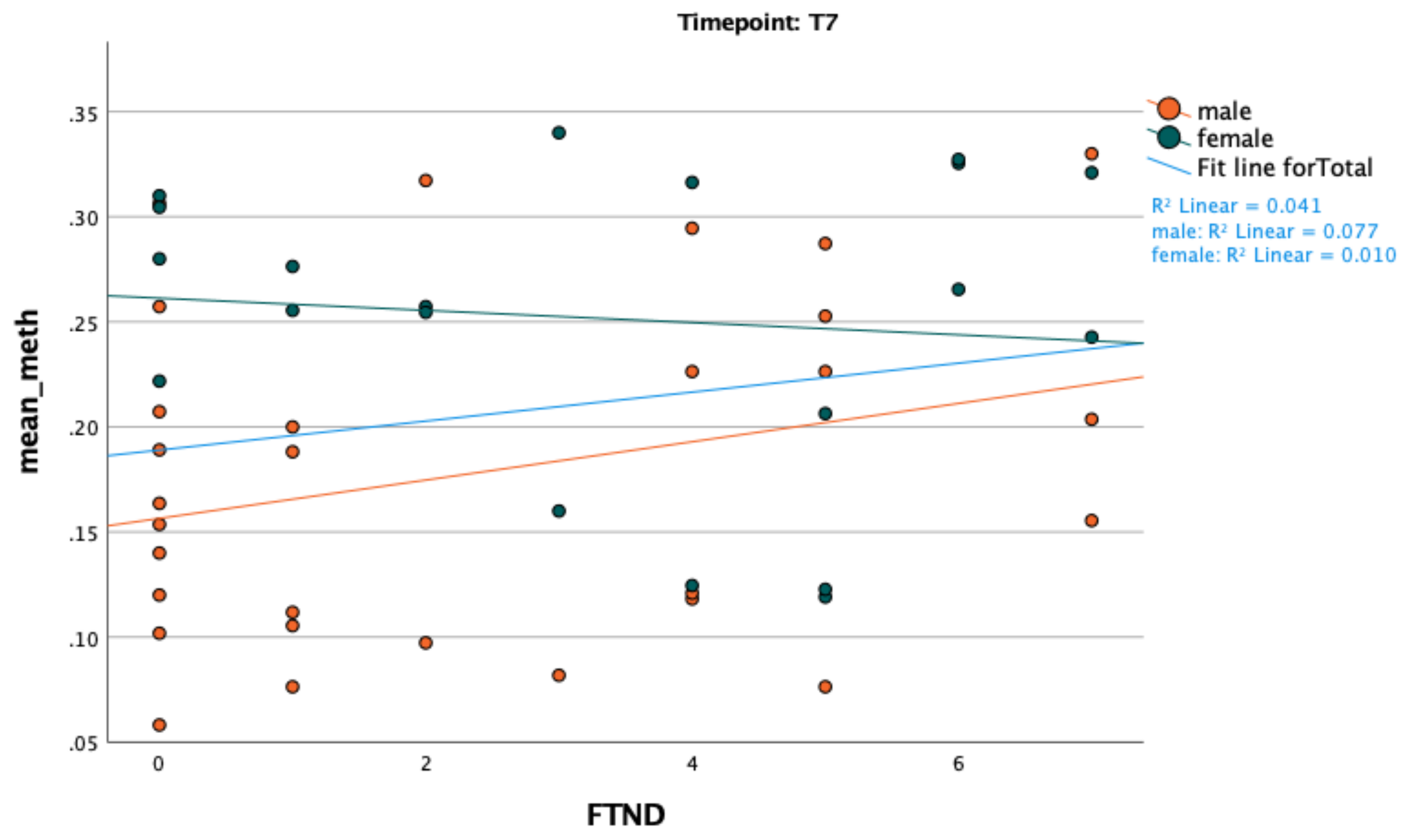

E

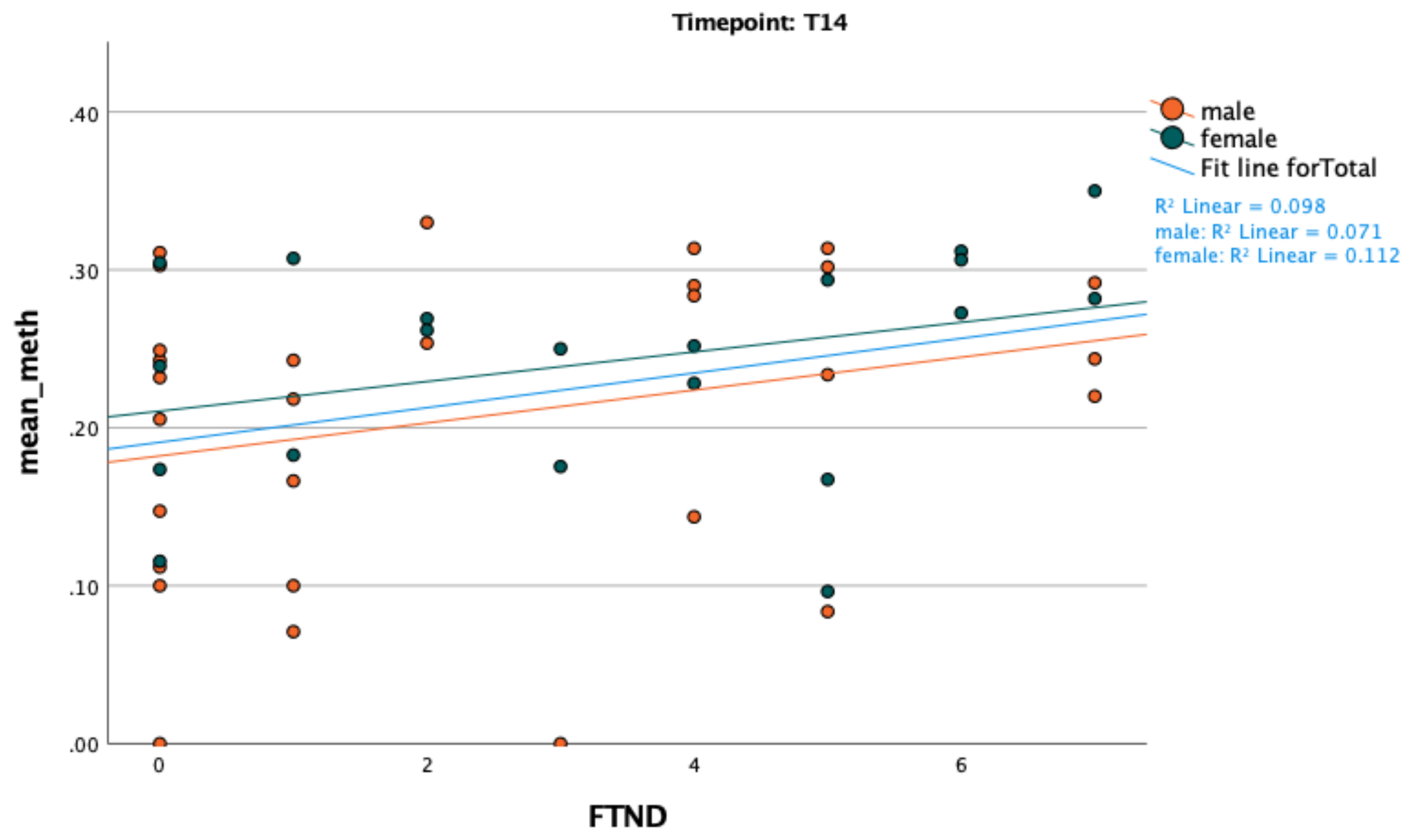

F

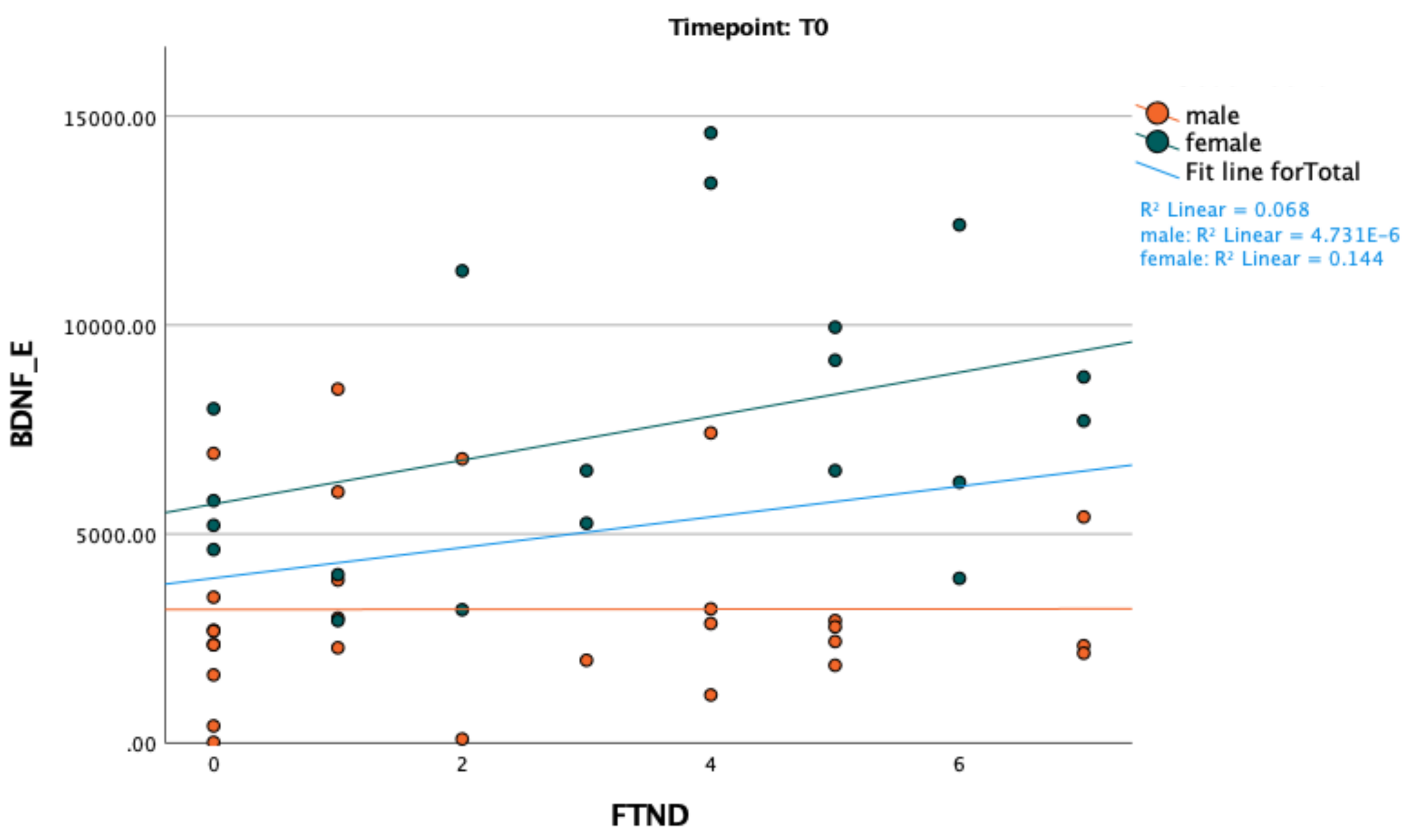

G

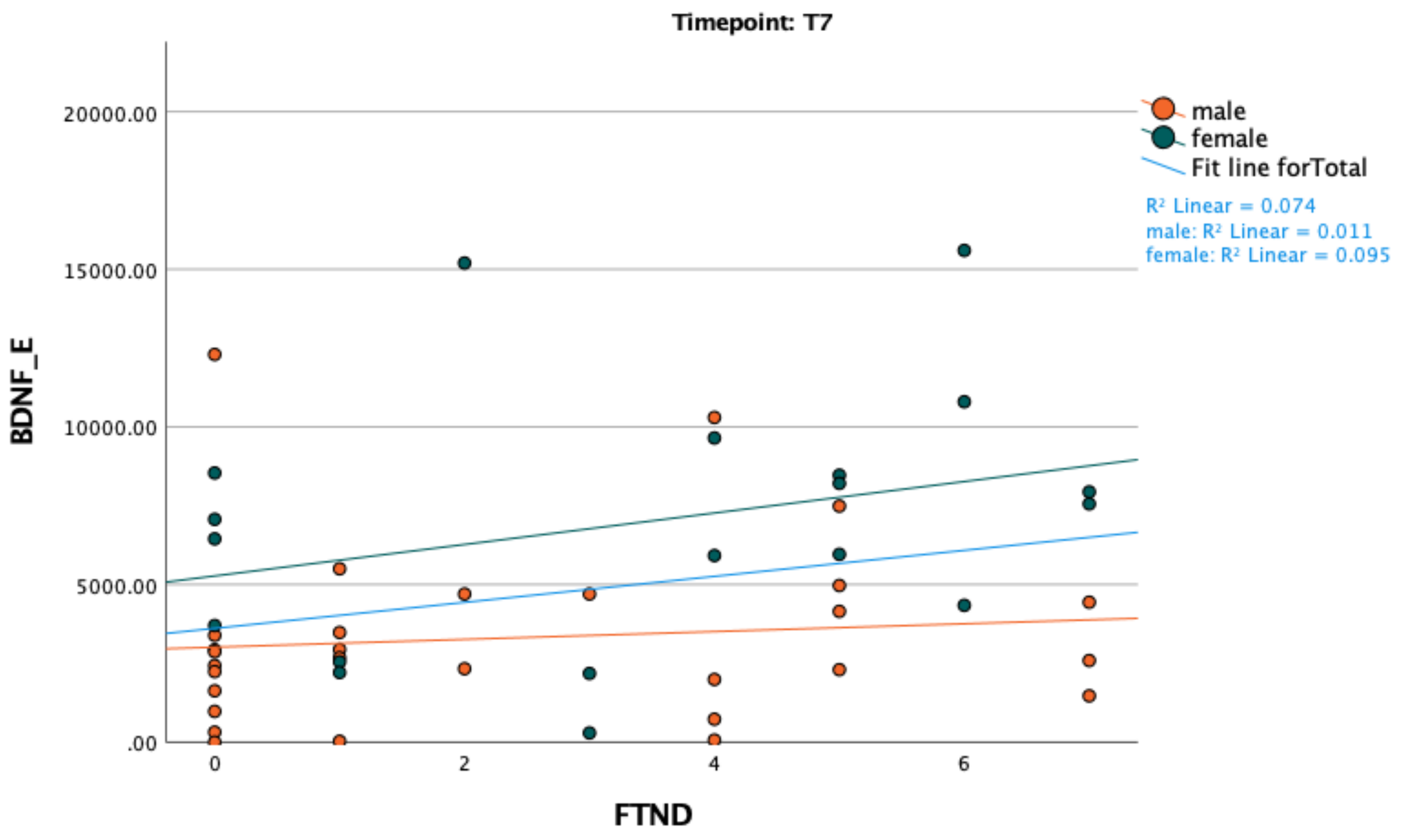

H

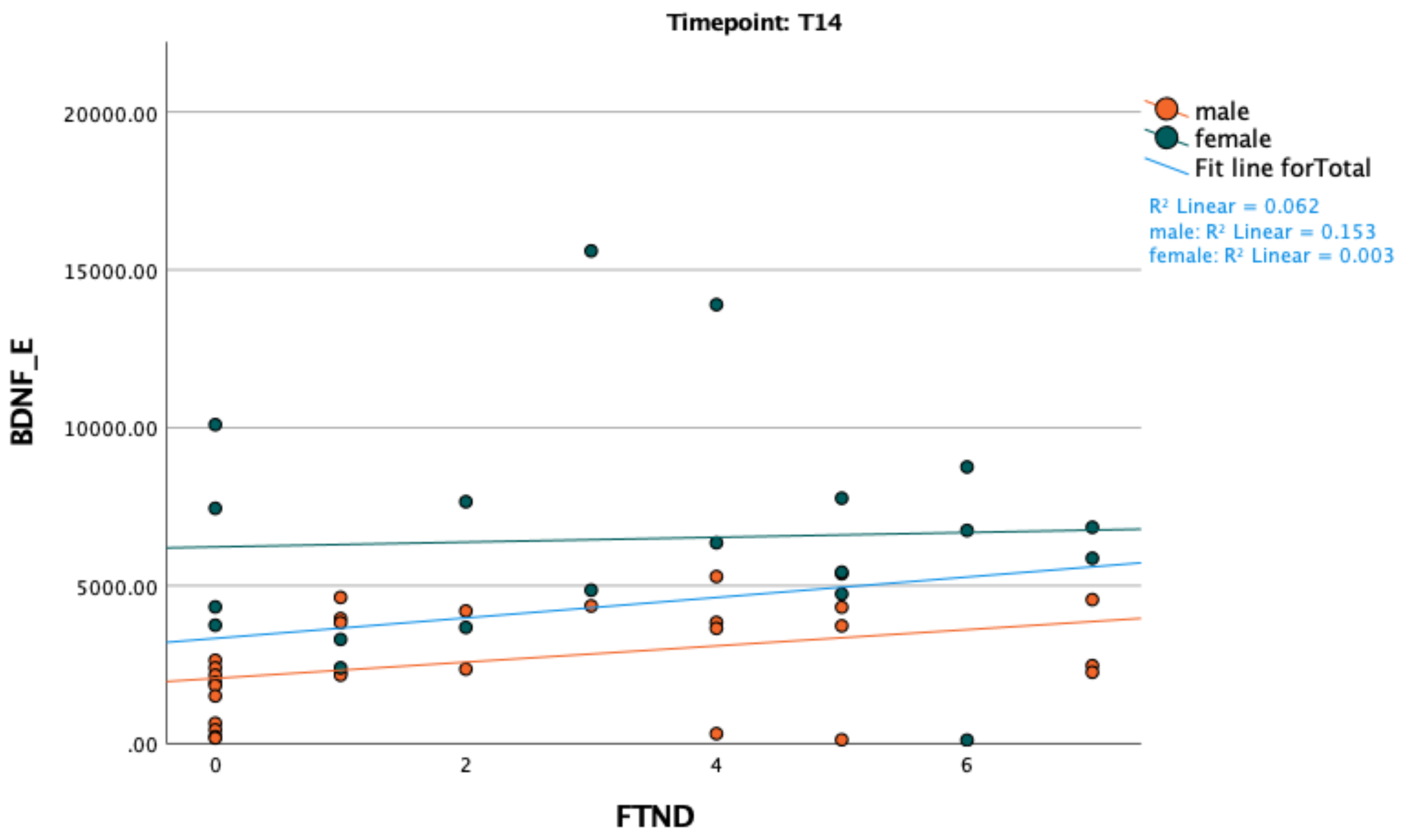

Supplement: Supplementary Figure S2 — Comparison of methylation and protein levels with the Fagerström questionnaire results (FTND). (A) Nonparametric Spearman Correlation of Protein levels (BDNF_E), mean methylation (mean_meth), FTND and FTND binning for groups defined by Lesch et al. (>3 = 1, 3–4 = 2, >4 = 3, FTND Lesch). (B) Display of mean methylation values for the three Lesch FTND groups divided by timepoint (T0-green, T7-dark red, T14-bright red). (C–H) Plot of mean methylation (C–E) and protein levels (F–H) against the FTND questionnaire results. Regression means with R square values for total trend and according to sex are given for each timepoint. [file Data_Sheet_2.PDF]
